# Research on Determining the Critical Influencing Factors of Carbon Emission Integrating GRA with an Improved STIRPAT Model: Taking the Yangtze River Delta as an Example

**DOI:** 10.3390/ijerph19148791

**Published:** 2022-07-19

**Authors:** Feipeng Guo, Linji Zhang, Zifan Wang, Shaobo Ji

**Affiliations:** 1 School of Management and E-Business, Zhejiang Gongshang University, Hangzhou 310018, China; guofp@mail.zjgsu.edu.cn (F.G.); 21020200029@pop.zjgsu.edu.cn (Z.W.); 2 Modern Business Research Center, Zhejiang Gongshang University, Hangzhou 310018, China; 3 Sprott School of Business, Carleton University, Ottawa, ON K1S 5B6, Canada; shaobo.ji@carleton.ca

**Keywords:** energy consumption, Yangtze River Delta, GRA, improved STIRPAT model, carbon emission influencing factors

## Abstract

Driven by China’s peak carbon emissions and carbon neutrality goals, each region should choose a suitable local implementation path according to local conditions, so it is of great significance to mine and analyze the critical influencing factors of regional carbon emissions. Therefore, this paper integrates grey relation analysis (GRA) and an improved STIRPAT model and selects the Yangtze River Delta region of China as the research object to analyze the factors affecting carbon emissions in four provinces in the region. Firstly, it uses the IPCC method to calculate the energy carbon emissions of each province. Secondly, according to the existing research, the relevant influencing factors of carbon emissions are sorted and summarized as candidate sets and this paper uses GRA to calculate the correlation degree of the above candidate sets. On this basis, this paper combines with the characteristics of the improved STIRPAT model to determine the index selection criteria and filter out the critical factors of each province. Thirdly, an improved STIRPAT model is constructed for each province to explore the influence of critical factors and analyze the influencing factors of carbon emissions in detail. The empirical results show that during the period from 2005 to 2019, the carbon emissions of the four provinces in the Yangtze River Delta are significantly different in structure and trend. At the same time, the critical influencing factors of each province are different and the influence of the same factor on different regions is significantly different. Finally, the policy suggestions for the provinces to achieve their peak carbon emissions and carbon neutrality goals are precisely tailored to the different carbon emission influencing factors.

## 1. Introduction

Global warming is becoming a serious challenge threatening the survival of human beings and the sustainable development of society. In the face of the increasingly serious climate change, how to effectively cope with and reduce carbon dioxide emissions has become an important social and environmental issue. As the world’s largest carbon emitting country, China is in the transition stage from high-speed growth to high-quality development and reducing carbon emissions and achieving low-carbon development are major strategic measures to build a green cycle of sustainable development [1,2]. In 2020, President Xi proposed at the 75th General Debate of the UN General Assembly that China will peak carbon emissions by 2030 and achieve carbon neutrality by 2060 (peak carbon emission and carbon neutrality goals). However, different provinces in China have different economic and population structures and achieving these peak carbon emissions and carbon neutrality goals requires a substantial transformation of the socio-economic and industrial structure at the national level on the one hand and targeted energy-saving and emission reduction strategies in each province and region on the other [3]. Therefore, strengthening research on the factors influencing carbon emissions in different provinces of China and analyzing regional differences in carbon emissions will effectively reduce greenhouse gas emissions, help achieve peak carbon emissions and carbon neutrality goals, and provide help to enhance the scientific and accurate energy conservation and emission reduction policies.

At present, domestic and foreign scholars have analyzed the influencing factors of carbon emissions from energy consumption from the perspectives of country, province and city. Firstly, at the national level, Haseeb et al. [4] tested the impact of urbanization, energy consumption and per capita GDP on carbon dioxide emissions in all BRICS (Brazil, Russia, India, China and South Africa) countries using panel data for 1990–2014; Shahbaz et al. [5] analyzed the impact of urbanization, prosperity and trade openness on Malaysia’s energy consumption. The results show that urbanization is the main factor affecting Malaysia’s energy consumption growth. Secondly, from a provincial perspective, Wang et al. [6] used the STIRPAT model to analyze the influencing factors of energy consumption in 30 provinces in China and found that when the population increased by 1%, the total national energy consumption increased by 1.992%; Huang et al. [7] studied the influencing factors of carbon emissions from energy consumption in Chongqing based on the STIRPAT model. Finally, from an urban perspective, Wang et al. [8] quantitatively determined the evolution path of urban carbon emissions in China; Qiu and Xu [9] conducted an empirical study on the spatial and temporal differences of carbon emissions in 13 representative cities of eight urban agglomerations in China; Wang et al. [10] used the super-efficient SBM model to measure urban carbon emissions performance. Although there are many studies on the influencing factors of carbon emissions, the results of different studies are quite different due to different research objects, methods and data obtained, and some conclusions are even contradictory. In addition, the driving forces of carbon emissions in different regions are different, so it is necessary to refine their influencing factors. However, a few scholars now comprehensively consider various influencing factors and conduct differentiated analysis according to the characteristics of different regions. Therefore, it is necessary to excavate appropriate variables from the multi-dimensional influencing factors to precisely analyze the influencing factors of carbon emissions in different regions.

As one of the regions with the highest level of economic development in China, the total energy consumption in the Yangtze River Delta region has remained at about 16% of the national proportion in the past 20 years and carbon emissions are in the forefront. There are differences in the development stage, industrial structure and prosperity of different provinces in the region. To this end, this study innovatively integrates the GRA algorithm and the improved STIRPAT model to analyze the differential energy carbon emission influencing factors of four provinces in the Yangtze River Delta region of China. On this basis, this study proposes targeted optimization strategies that will contribute to the high-quality, green and sustainable socio-economic development of the Yangtze River Delta region and the achievement of peak carbon emissions and carbon neutrality goals, with important theoretical and practical significance.

The rest of this paper is organized as follows: Section 2 introduces the related works of the study; Section 3 outlines the methodology; Section 4 states the data sources and conducts the empirical analysis; and Section 5 discusses the results. Finally, Section 6 draws the conclusion and Section 7 makes targeted policy recommendations.

## 2. Related Works

### 2.1. Study on Influencing Factors of Carbon Emissions

The carbon emission system is an open and complex system. Studies have shown that there are many factors affecting carbon emissions, such as changes in population, economy and technology, among other fields that also have a significant impact on carbon emissions [11].

In terms of population, the evolution of population size and population urbanization can have an impact on carbon emission processes. Early studies have confirmed that population size and urbanization are key factors affecting carbon dioxide emissions in China and regions above the provincial level [12,13]. Fan et al. [14] analyzed the impacts of population on the total carbon dioxide emissions of countries with different income levels from 1975 to 2000 and found that the impacts of population on carbon dioxide are different at different development levels. Wang [15] explored the relationship between population urbanization and carbon emissions based on cross-country panel data; the results show that the relationship between the two is inverted U-shaped. Shahbaz et al. [5] pointed out that urbanization is an important factor affecting carbon dioxide emissions.

In terms of economic and social development, the impacts on carbon emissions are mainly explored at the level of economic development, industrial structure and foreign investment. Using data from 1978 to 2006, Lin et al. [16] analyzed the impact of per capita GDP and industrialization on the national environment. The results showed that both of two factors were found to have a significant effect on carbon emissions. Liu [17] explored the factors influencing carbon emissions in six provinces in central China and the results showed that economic growth is the dominant factor in the growth of carbon emissions. Zhang [18] concluded that the tertiary industry is also an important source of carbon emissions: industrial structure affects the total energy consumption, thereby affecting carbon dioxide emissions. Foreign direct investment (FDI) is becoming the main source of carbon emissions as globalization accelerates and trade between countries becomes more frequent. By using the panel data of five ASEAN countries, Baek [19] found that foreign direct investment tends to increase carbon emissions. Zhang et al. [20] studied the impact of FDI on China’s carbon emissions and pointed out higher FDI associated with higher emissions. Omri et al. [21] used the dynamic simultaneous equations panel data model to investigate the causal relationship between carbon emissions and FDI in 54 countries and provided evidence of a two-way causal relationship. Xu [22] found that enhancing the level of foreign trade can reduce per capita carbon emissions to a certain extent. Yao [23] believes that foreign direct investment can effectively reduce carbon intensity everywhere.

In terms of technology level, energy intensity and carbon emission intensity are mainly used as a representation of technological progress. Lin [24] decomposed the factors influencing carbon emissions in China’s chemical industry based on the LMDI method and the results showed that energy intensity is the main factor inhibiting carbon emissions. Li [25] found that the reduction in carbon emission intensity in a region not only directly reduces its own carbon emission level, but also has a positive spatial spillover effect on neighboring regions.

In addition to the above factors, in recent years, scholars have included more potential factors in the study of carbon emissions. By analyzing the indirect carbon emissions of China’s residential consumption, Yuan [26] found that the driving role of consumption level and consumption structure on carbon emissions cannot be ignored. For the first time, Li [27] included the number of industrial enterprises above scale in the analysis of carbon emission factors and the results showed a significant effect on carbon emissions in some regions.

### 2.2. Study on Methods of Carbon Emission Analysis

In the grey system theory, a system with completely clear information is called a white system, a system with incomplete information is called a black system, and a system with partially clear information and uncertainty is called a grey system. Grey system theory modeling is mainly based on social, economic and technological factors to find the statistical relationship between various factors [28] and is now widely used in the field of carbon emission. Lottfalipour et al. [29] compared the prediction performance of the grey model and ARIMA and found that the grey system can achieve higher accuracy in carbon emission prediction. Pao and Tsai [30] apply grey models to predict pollutant emissions, energy consumption and production in Brazil. Ma et al. [31] searched for influencing factors and analyzed their joint impact on carbon emissions from a spatial and temporal perspective by means of association rule algorithms.

In the study of carbon emission influencing factors-related methods, commonly used methods include the environmental Kuznets curve [32,33], IPAT equation [34,35,36], LMDI decomposition method [37,38], STIRPAT equation [39,40,41,42,43], BP neural network [44,45], system dynamics [46,47], etc. Among them, the STIRPAT model is an extensible random environmental impact assessment model widely used in carbon emission research [48]. In recent years, many scholars have expanded the traditional STIRPAT model from the perspectives of urbanization, trade and investment and environmental pollution and the superposition of various methods has made the research more scientific. Liu et al. [49] introduced spatio-temporal weighting factors into the STIRPAT model to analyze carbon dioxide emission factors in Chinese provinces; Yang and Liu [50] introduced factors such as trade and investment into the traditional model to analyze the influencing factors of inter-regional emission differences in China; Li et al. [51] explored the driving forces affecting CO_2_ emissions in China based on a model combining path analysis and STIRPAT; Li et al. [27] analyzed the influencing factors of carbon emissions by principal component analysis and stepwise regression analysis; Song et al. [37] applied LMDI factor decomposition and STIRPAT model to empirically analyze the drivers of energy carbon emissions in the Yangtze River Delta region.

In summary, many scholars have carried out a substantial amount of research on the influencing factors of carbon emissions in different regions from multiple perspectives and achieved fruitful results. However, the following problems still exist: Firstly, some studies only focus on single influencing factors, ignoring the complex relationship between different factors, making it difficult to fully feedback the driving forces of regional carbon emissions. Secondly, considering too many factors comprehensively, all factors are put into the model for analysis, which affects the accuracy and stability of the results. It is necessary to eliminate the model variables and optimize the model structure. Therefore, this paper applies the GRA method to explore the deep relationship between carbon emission and its influencing factors, constructs improved STIRPAT models based on the critical influencing factors and uses the results of each model to precisely analyze the influencing factors of carbon emissions in the target area, so as to put forward an optimization strategy for realizing the peak carbon emission and carbon neutrality goals.

## 3. Methods

**Figure 1:**
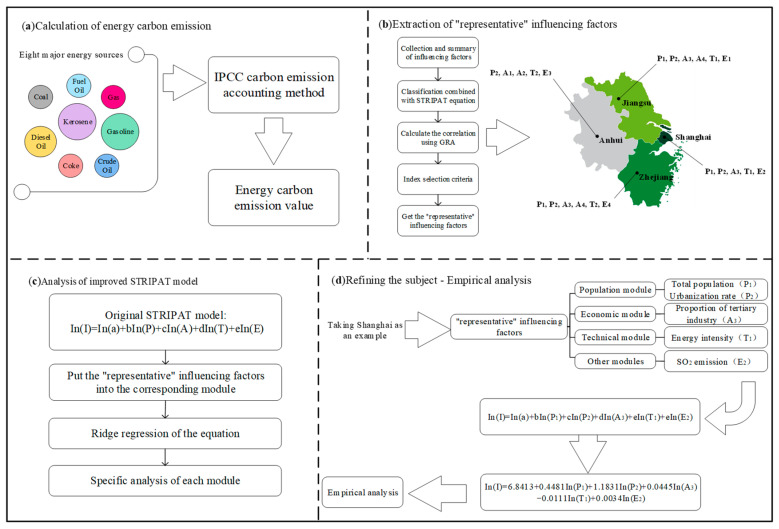
Method flow chart.

This paper takes four provinces (Anhui, Jiangsu, Shanghai and Zhejiang) in the Yangtze River Delta region of China as the research objects. Firstly, the IPCC method is used to calculate the energy carbon emissions of each province. Secondly, according to the existing research, the influencing factors of carbon emissions are summarized as the candidate set. Thirdly, the GRA algorithm is used to calculate the correlation degree of the above candidate set. On this basis, the index selection criteria are determined by combining the characteristics of the STIRPAT equation to filter the critical influencing factors of each province. Finally, an improved STIRPAT model is constructed for each province to explore the influence of critical factors and analyze the influencing factors of carbon emissions in detail. The overall flow of the method in this paper is shown in Figure 1.

### 3.1. Carbon Emission Calculation

An inevitable problem in the empirical study of carbon emission factors is the measurement of carbon emission. Due to the lack of direct monitoring statistics, the carbon emission measurement of the research object will directly affect the accuracy of the empirical results. This paper uses fossil energy as the carbon source to measure the carbon emission of each province and the most widely used carbon emission coefficient method is used at present. The calculation of energy carbon emissions is carried out according to the IPCC accounting framework:

(1) $$C=\overset{8}{\underset{i=1}{\sum }}E_{i}F_{i}K_{i}\times \frac{44}{12}$$

In the formula: C is the total carbon emission caused by energy; E is the energy consumption; i represents the corresponding energy types, such as coil, coke, crude oil, etc.; and F_i_ and K_i_ represent the standard coal conversion factor and carbon emission factor for type i energy. Various energy coefficients used in this accounting framework are referenced from the “Guidelines for Compiling Provincial Greenhouse Gas Inventories” [52]. The specific values are shown in Table 1.

### 3.2. Analysis of Influencing Factors of Carbon Emissions

Based on the literature review of the factors influencing carbon emissions in Section 2.1, 11 relevant factors were identified. In addition, SO_2_ emissions are considered to be closely related to greenhouse gas emissions [53]; with the improvement of living standards, the number of private cars has increased significantly and the carbon emissions of private trips are five times higher than those of public transportation [54]. Based on this, this study included SO_2_ emissions and civil vehicle ownership in the analysis and finally aggregated and identified 13 influencing factors related to carbon emissions as candidates. In order to further explore the relationship between carbon dioxide emissions and various influencing factors, based on the relevant data compiled and calculated, combined with the STIRPAT equation in Section 3.4, the 13 variables were classified into four modules: population factors, economic development, technological factors and other factors. All the influencing factors are shown in Table 2.

### 3.3. GRA of Carbon Emission

As part of grey system theory, GRA is very effective in solving problems with complex interrelationships between multiple factors and variables [55]. The GRA model was first developed by Deng [28] and provides an impact assessment model that can calculate the similarity between each potential impact factor and the reference sample. Specifically, combined with the context of this paper, the relationship between the influencing factors of carbon emissions based on GRA is analyzed as follows:

Step 1: Construct the initial decision matrix X, assuming there are n data sequences and m candidate factors, the j-th value under the i-th candidate factor is represented as x_ij_. In this paper, the candidate factors are the influencing factors in Section 3.2, such as total population, per capita GDP, energy intensity, etc. The data sequence is Anhui, Jiangsu, Shanghai, and Zhejiang and the initial matrix X = {x_ij_}_m×n_ can be obtained:

(2) $$X=\left[\begin{array}{cccc} x_{11} & x_{12} & \cdots & x_{1n}\\ x_{21} & x_{22} & \cdots & x_{2n}\\ \vdots & \vdots & \ddots & \vdots \\ x_{m1} & x_{m2} & \cdots & x_{\mathrm{mn}} \end{array}\right]$$

Step 2: Reduce the difference in absolute value between the data of different carbon emission influencing factors and normalize the initial matrix X:

(3) $$x_{i}^{\prime }\left(j\right)=\frac{x_{i}\left(j\right){-\min}_{i=1}^{n}\left[x_{i}\left(j\right)\right]}{\max_{i=1}^{n}\left[x_{i}\left(j\right)\right]{-\min}_{i=1}^{n}\left[x_{i}\left(j\right)\right]}$$

Generate normalized matrix X′:

(4) $$X^{\prime }=\left[\begin{array}{cccc} x^{\prime }{}_{11} & x^{\prime }{}_{12} & \cdots & x^{\prime }{}_{1n}\\ x^{\prime }{}_{21} & x^{\prime }{}_{22} & \cdots & x^{\prime }{}_{2n}\\ \vdots & \vdots & \ddots & \vdots \\ x^{\prime }{}_{m1} & x^{\prime }{}_{m2} & \cdots & x^{\prime }{}_{\mathrm{mn}} \end{array}\right]$$

Reference sequence:

(5) $$x_{0}^{\prime }{=x}_{0}^{\prime }\left(1\right){,x}_{0}^{\prime }\left(2\right){,\ldots ,x}_{0}^{\prime }\left(m\right)$$

where $x_{0}^{\prime }\left(j\right)$ is the reference value relative to the j-th data obtained after processing the reference column (this article is carbon emissions).

Step 3: Calculate the difference between each sample value and the reference value and construct a difference matrix:

(6) $$\Delta_{0i}\left(j\right)=\left|x_{0}^{\prime }\left(j\right)\;-\;x_{i}^{\prime }\left(j\right)\right|$$

(7) $$\Delta =\left[\begin{array}{cccc} \Delta_{01}\left(1\right) & \Delta_{01}\left(2\right) & \cdots & \Delta_{01}\left(m\right)\\ \Delta_{02}\left(1\right) & \Delta_{02}\left(2\right) & \cdots & \Delta_{02}\left(m\right)\\ \vdots & \vdots & \ddots & \vdots \\ \Delta_{0n}\left(1\right) & \Delta_{0n}\left(2\right) & \cdots & \Delta_{0n}\left(m\right) \end{array}\right]$$

Step 4: Calculate the grey relation coefficient:

(8) $$\gamma_{0i}\left(j\right)=\frac{\min_{i=1}^{n}\min_{j=1}^{n}\Delta_{0i}\left(j\right){+\rho \times \max}_{i=1}^{n}\max_{j=1}^{m}\Delta_{0i}\left(j\right)}{\Delta_{0i}\left(j\right){+\rho \times \max}_{i=1}^{n}\max_{j=1}^{m}\Delta_{0i}\left(j\right)}$$

In the formula, ρ(0≪ρ≪1) is the discrimination coefficient and the smaller the ρ, the higher the distinguishability. By default, ρ is taken as 0.5, which has a moderate recognition effect and good stability.

### 3.4. Improved STIRPAT Model

The STIRPAT model is a random special form proposed by York and Dietz [56] on the basis of the IPAT identity. It examines the individual effects of population, wealth and technological factors on the environment when they change and eliminates the influence of the same proportional change problem. This method is currently the most commonly used method to study carbon emission peaks and has good scalability. Therefore, after filtering out the critical influencing factors of each province in GRA, this paper uses the improved STIRPAT expansion model to study the influencing factors of carbon emissions. The basic form of the model is:

(9) $${I=\mathrm{aP}}^{b}A^{c}T^{d}e$$

In the formula: I, P, A, T—Environmental pressures, population factors, wealth factors and technological levels; a—model coefficient; b, c, d—Elasticity coefficients of population, wealth, and technology; e—Error.

When the model is used in this paper to analyze the influencing factors of carbon dioxide emissions, I is used to represent the carbon emissions of a region. After taking the logarithm of both sides of the formula, we can obtain the formula:

(10) lnI=lna+blnP+clnA+dlnT+lne

In the formula: lna is a constant term: lne is a random distractor.

Due to its flexibility and openness, the STIRPAT model can dynamically adjust the control factors according to the research purpose and needs of this paper. Therefore, in this study, according to the results of GRA, models were constructed for the significant influencing factors of each province to analyze the specific influencing factors of carbon emissions in different provinces.

## 4. Empirical Analysis

### 4.1. Experimental Data

The available data were derived from different indicators. In this study, the original data of the 10 indicators of total population, urbanization rate, per capita GDP, proportion of the secondary industry, proportion of the tertiary industry, number of industrial enterprises above scale, SO_2_ emissions, per capita disposable income, per capita consumption expenditure and civil vehicle ownership are directly derived from the annual data of provinces in the China Statistical Yearbook from 2005 to 2019. The carbon emission index data are calculated from the consumption of eight major fossil fuels combined with the IPCC measurement algorithm, of which the consumption of fossil fuels comes from the China Energy Statistical Yearbook. The data of carbon emission intensity and energy intensity are calculated according to the energy carbon emission and fossil energy consumption of each province, converted into standard coal consumption and the total provincial GDP after conversion. The data of the foreign direct investment (FDI) indicator come from the respective statistical yearbooks of the four provinces.

### 4.2. Carbon Emission Calculation and Analysis

**Figure 2:**
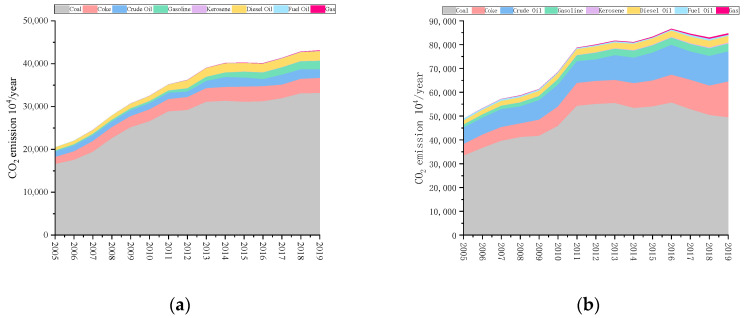

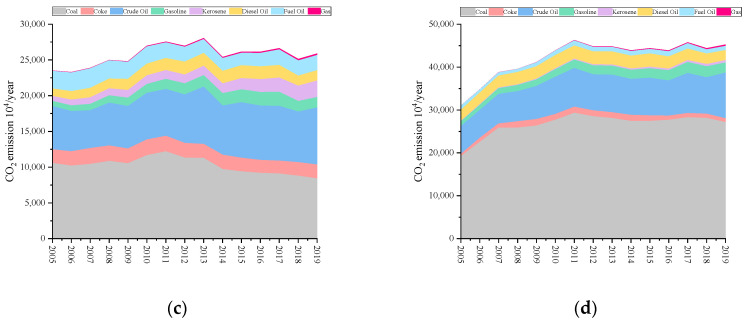
Energy carbon emission structure of each province from 2005 to 2019: (**a**) Anhui; (**b**) Jiangsu; (**c**) Shanghai; (**d**) Zhejiang.

**Figure 3:**
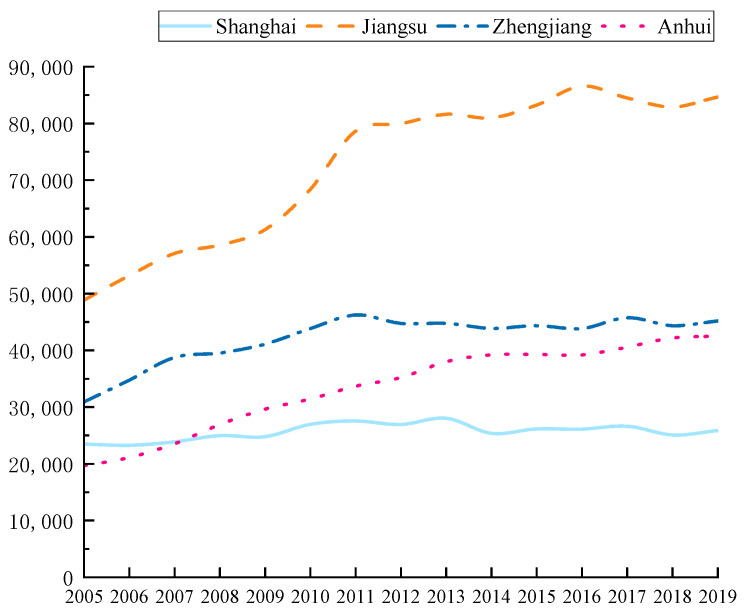
Total carbon emissions of provinces in the Yangtze River Delta region from 2005 to 2019.

According to the calculation method in Section 3.1, the energy carbon emissions of the four provinces in the Yangtze River Delta region from 2005 to 2019 are shown in Figure 2. It can be found that during the period from 2005 to 2019, the energy carbon emissions of the four provinces in the Yangtze River Delta region showed significant differences in structure and trend. Due to the early start of Shanghai’s urbanization process, carbon emissions fluctuated slightly in the past 15 years, while the total carbon emissions of the other three provinces have increased significantly. Among them, Jiangsu Province, which has the largest increment, increased from 488.4908 million tons in 2005 to 846.5748 million tons in 2019. From 2005 to 2011, except for Shanghai, the growth rate of carbon emissions in the three other provinces was relatively fast and then the two provinces of Jiangsu and Zhejiang began to slow down. In 2011, Zhejiang Province put forward the goal of energy conservation and emission reduction during the “Twelfth Five-Year Plan” period, which was fully completed in 2015 and the carbon emissions showed a corresponding downward trend. Compared with the other three provinces, Anhui Province is relatively backward in terms of economic level and is still in the stage of rapid development and carbon emissions have maintained a sustained high growth. The comparison of total carbon emissions in the four provinces is shown in Figure 3.

### 4.3. GRA of Carbon Emission Influencing Factors

**Figure 4:**
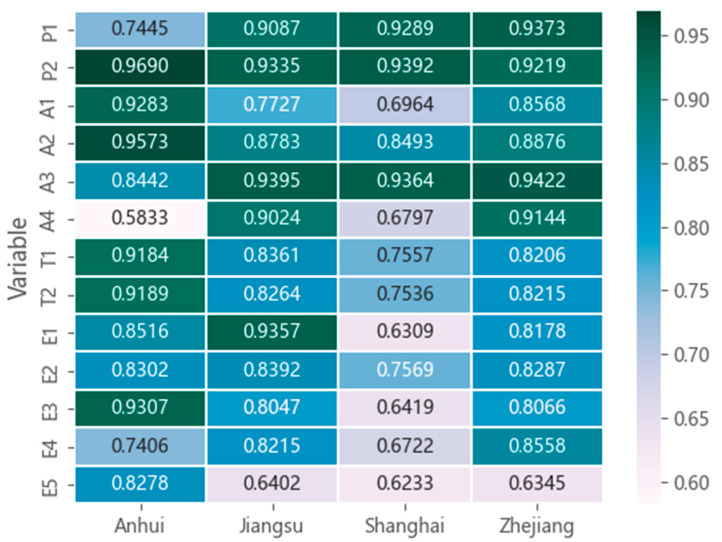
Grey correlation coefficient heatmap.

Using the GRA method, the correlation coefficients between the 13 influencing factors and carbon emissions were calculated. The results are shown in Figure 4. According to the results of GRA, it is found that $\gamma_{0i}{(j)\min=\gamma }_{06}(1)=0.5833$, $\gamma_{0i}{(j)\max=\gamma }_{02}(1)=0.9690$, There are 18 correlation coefficients $\gamma_{0i}\left(j\right)$ ≧ 0.9. It shows that the carbon emission influencing factor variables proposed in this paper have a good correlation with energy carbon emissions. In addition, although they are all located in the Yangtze River Delta region, the correlation coefficients between energy carbon emissions and driving factors in the four neighboring provinces are quite different, indicating that the main driving factors behind carbon emissions in each province are different.

In order to obtain the critical influencing factors, this study formulated the following selection criteria: First select the factor with the highest correlation coefficient from each module in each province and then set the threshold α = 0.9, In the remaining variables, if $\gamma_{0i}\left(j\right)$ ≧ α, then this factor will also be included in the final model. In addition, since energy intensity (T_1_) and carbon emission intensity (T_2_) are highly consistent, only the higher of the two factors is selected here. Taking Shanghai as an example here, the selection process is as follows:

1. Select the highest score for each module among the four modules of economic development, population factors, technological factors and other factors: these are the urbanization rate (P_2_), the proportion of the tertiary industry (A_3_), energy intensity (T_1_), and SO_2_ emissions (E_2_);
2. In the remaining variables, find the $\gamma_{01}\left(3\right)=0.9289≧\alpha$. Therefore, taking the total population (P_1_) as the screening result, the screening ends.

**Figure 5:**
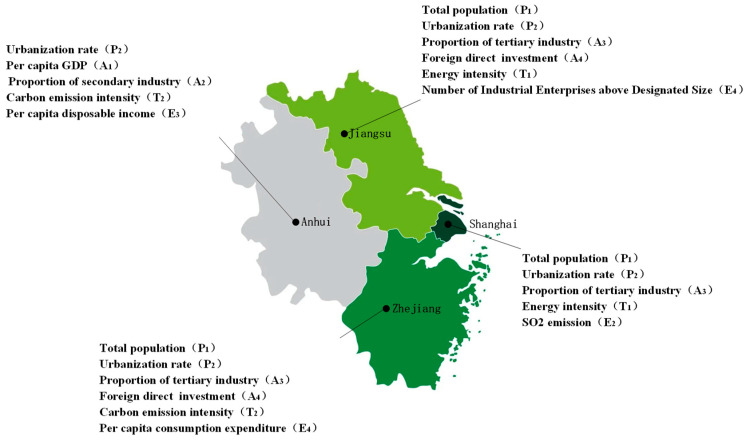
Critical influencing factors selection result chart.

According to the above criteria, the final critical influencing factors selection results of each province are shown in Figure 5.

### 4.4. Improved STIRPAT Model Analysis

According to the results of GRA in Section 4.2, taking carbon emissions as the explained variable, the critical carbon emission influencing factors were selected as the explanatory variables and the improved STIRPAT models corresponding to different provinces were constructed as shown in Table 3.

In order to avoid the multicollinearity interference of the panel data, this study uses the ridge regression method to regress the data to retain the information of the independent variables and dependent variables to a greater extent. Calculate the equations, ridge traces, and goodness of fit R^2^ corresponding to different ridge parameter K values. The smaller the K, the less information the sample data lose and the higher the model accuracy. Through prior analysis, it is found that when K = 0.2, the coefficients of each model are relatively stable. In order to facilitate unified comparison, this study finally determined the value of K to be 0.2. Under this value, this study carried out a regression analysis on four models; the effects of each model are shown in Table 4. The experimental results explain the contribution of each critical influencing factor of carbon emission in the regression model. The R-Square of the four models is the lowest of 0.7007 in Shanghai, indicating that the factors in the model explain 70.07% of the carbon emission factors; the remaining 29.93% of the influencing factors are not included in the model. Anhui with the highest R-Square value is 0.9872, which has excellent explanatory power.

The model results in Anhui are shown in Table 5. The improved STIRPAT model obtained from the results is Equation (11). From the fitting results of the model, it can be seen that the order of the effect of the influencing factors is as follows: A_2_ > P_2_ > T_2_ > E_3_ > A_1_. Except for carbon emission intensity (T_2_), which is negatively correlated, all other factors are positively correlated with total carbon emissions. The proportion of secondary industry is the strongest factor for the growth of carbon emissions from energy consumption in Anhui Province and the elasticity coefficient is 0.5748, indicating that for every 1% increase in the proportion of secondary industry in Anhui Province, the total carbon emissions will increase by 0.5748%. Compared with the other three provinces, the economic development of Anhui Province has a low starting point and a late start. By accelerating the industrialization process, the economic level has been improved, which has resulted in excessive energy consumption and a large amount of carbon emissions. For every 1% increase in urbanization rate (P_2_), per capita disposable income (E_3_) and per capita GDP (A_1_), total carbon emissions will increase by 0.3804%, 0.1187% and 0.1025%, respectively. The growth of urbanization rate is accompanied by the improvement of residents’ living standards and the vigorous development of real estate, transportation and other industries. These interrelated factors have jointly promoted the increase in carbon emissions in Anhui Province. It is worth noting that the elasticity coefficient of carbon emission intensity (T_2_) is −0.1341; that is, for every 1% increase in T_2_, the total carbon emission of energy consumption will decrease by 0.1341%. The higher the carbon emission intensity, the smaller the proportion of carbon consumption in the unit output value of the region. Therefore, improving the level of production technology will be one of the effective ways to reduce carbon dioxide emissions in the region.

(11) $${\mathrm{lnI}}_{1}=9.0179+0.3804{\mathrm{lnP}}_{2}+0.1025{\mathrm{lnA}}_{1}+0.5748{\mathrm{lnA}}_{2}-\;0.1341{\mathrm{lnT}}_{2}+0.1187{\mathrm{lnE}}_{3}$$

Table 6 shows the regression results of Jiangsu Province. As the province with the largest volume among the four provinces, the influence degree of Jiangsu Province is: P_1_ > P_2_ > A_3_ > A_4_ > E_1_ > T_1_. Population factors become the most influential module. Every 1% increase in the total population (P_1_) and urbanization rate (P_2_) will cause carbon emissions to increase by 1.1761% and 0.5128%, respectively. For every 1% increase in the proportion of the tertiary industry (A_3_) and foreign direct investment (A_4_), the total carbon emissions will increase, respectively, by 0.3225% and 0.2094%. For every 1% increase in the number of industrial enterprises above scale (E_1_) and energy intensity (T_1_), the total carbon emissions will be reduced by 0.0593% and 0.0571%, respectively. The special economic and social development stage of Jiangsu Province causes this particularity. As a strong economic province in China, its economic development rate is at the forefront of the country and it has undertaken a large number of industries transferred from Shanghai, attracted a large number of outstanding talents and has received a significant foreign population. In addition, the speed of industrial transformation in Jiangsu Province is relatively fast. In the past 15 years, the proportion of the tertiary industry has increased from 35.85% to 51.54%, which is consistent with the growth trend of total carbon emissions.

(12) $${\mathrm{lnI}}_{2}=0.6335+1.1761{\mathrm{lnP}}_{1}+0.5128{\mathrm{lnP}}_{2}+0.3225{\mathrm{lnA}}_{3}+0.2094{\mathrm{lnA}}_{4}-0.0571{\mathrm{lnT}}_{1}-\;0.0593{\mathrm{lnE}}_{1}$$

Table 7 shows the model fitting results of Shanghai. The degree of influence of the critical influencing factors in Shanghai is in the order of P_2_ > P_1_ > A_3_ > T_1_ > E_2_. Population factors have the most significant impact on total carbon emissions. For every 1% increase in urbanization rate (P_2_) and total population (P_1_), total carbon emissions will increase by 1.1831% and 0.4481%. Shanghai is the largest economic center city in China, with a high level of urbanization, large urban scale, long average travel distance, and long daily electricity consumption for living and business. As the population grows, the demand for goods and services increases significantly, leading to rising carbon emissions. Relatively speaking, the coefficients of other factors are relatively low. For every 1% increase in the proportion of the tertiary industry (A_3_) and SO_2_ emissions (E_2_), carbon emissions will increase by 0.0445% and 0.0034%, respectively, and the energy structure (T_1_) will decrease by 0.0111%. Affected by this policy, Shanghai’s energy-intensive enterprises have completed a large number of transfers. At the same time, the tertiary industry has developed rapidly: from 52.13% in 2005 to 72.88% in 2019. The tertiary industry such as financial insurance, real estate and social services has achieved rapid development. These industries have low carbon emission intensity, further highlighting the impact of population factors on total carbon emissions.

(13) $${\mathrm{lnI}}_{3}=6.8413+0.4481{\mathrm{lnP}}_{1}+1.1831{\mathrm{lnP}}_{2}+0.0445{\mathrm{lnA}}_{3}-\;0.0111{\mathrm{lnT}}_{1}+0.0034{\mathrm{lnE}}_{2}$$

The model fitting results of Zhejiang Province are shown in Table 8 and the order of influence is as follows: P_2_ > E_4_ > A_3_ > A_4_ > P_1_ > T_2_. Urbanization rate (P_2_) is the most significant factor affecting Zhejiang’s carbon emissions. Every 1% increase will bring a total of 1.2537% of the total carbon emissions; per capita consumption expenditure (E_4_), foreign direct investment (A_4_) and total population (P_1_) is positively correlated and the elastic coefficients are 0.5603, 0.0980, and 0.0640, respectively. The proportion of tertiary industry (A_3_) and carbon emission intensity (T_2_) are negatively correlated with total carbon emissions and each increase of 1% will decrease by 0.1277% and 0.0018%. The level of urbanization is also the most significant influencing factor in Zhejiang and the coefficient of influence on carbon emissions is significantly positive, which is consistent with previous research conclusions [57,58]. In the process of urbanization, production activities have increased, the frequency and scope of transportation have increased and the large-scale construction of urban infrastructure, the development of real estate and increased energy consumption have resulted in a large amount of carbon dioxide. For the first time, per capita consumption expenditure (E_4_) has become the critical influencing factor of regional carbon emissions. This is because the people of Zhejiang Province are relatively affluent and the regional consumption capacity is strong, resulting in huge demand for energy consumption. Since the reform and opening-up, foreign direct investment has also become an important driving force for China’s economic development. Zhejiang is located on the coast with a high level of opening to the outside world and advanced development concepts, attracting a large number of foreign investments to build factories, which have become a major factor in the growth of total carbon emissions.

(14) $${\mathrm{lnI}}_{4}=9.6033+0.0640{\mathrm{lnP}}_{1}+1.2537{\mathrm{lnP}}_{2}-\;0.1277{\mathrm{lnA}}_{3}+0.0980{\mathrm{lnA}}_{4}-\;0.0018{\mathrm{lnT}}_{2}+0.5603{\mathrm{lnE}}_{4}$$

According to the empirical results, it can be seen that there are significant differences in the influencing factors of carbon emissions in different regions. The total energy carbon emissions in Shanghai and Zhejiang are most affected by urbanization rate; Jiangsu is the total population and Anhui is the proportion of secondary industry in the economic development factors. In addition to industrial structure, among economic development factors, FDI has become the critical influencing factor of Jiangsu and Zhejiang and per capita GDP has become the critical influencing factor of Anhui Province. Compared with other critical factors, technical factors have lower elastic coefficients in all four models. In the other factors module, the critical factors of the four provinces are different and have different degrees of influence on the total carbon emissions. The most notable is the per capita consumption expenditure of Zhejiang Province with an elasticity coefficient of 0.5603, which, to a certain extent, is consistent with Zhejiang Province being the first demonstration area of common prosperity in China.

**Figure 6:**
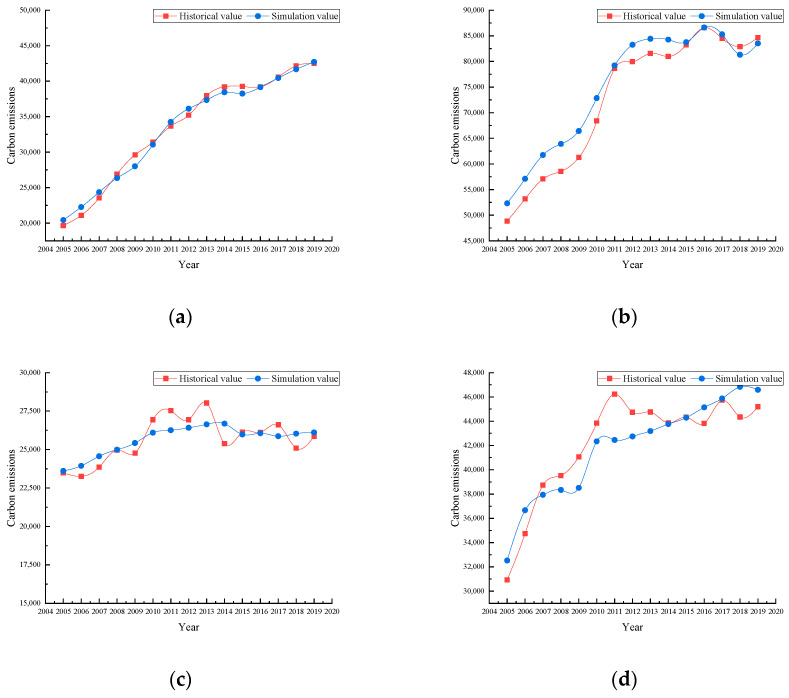
The contrast of historical and simulation carbon emissions value of each province from 2005 to 2019: (**a**) Anhui; (**b**) Jiangsu; (**c**) Shanghai; (**d**) Zhejiang.

Finally, according to the real data of each factor, the four models constructed in this paper are used to simulate the carbon emissions of each province and the simulated values and historical values are regressed. The results show that the simulation effect is good. The comparison between the simulation value and the historical value is shown in Figure 6.

## 5. Discussion

This study empirically analyzes the structure of regional differences in carbon emissions among four provinces in the Yangtze River Delta region of China, determines the critical influencing factors of carbon emissions in the four provinces and reveals the regional heterogeneity of carbon emissions. The results show that the model has good stability and fitting effect (Table 4, Figure 6). The paper provides an empirical basis for the regional development of low-carbon emission reduction policies and the model is equally applicable to other regions. This research differs from other achievements; for example, Xu revealed the existence of EKC for provincial carbon emissions in China [59] but lacked an analysis of the influencing factors. Although Zhao et al. [60] analyzed the factors influencing carbon emissions, their analysis of specific factors focused only on aspects such as urbanization and industrial structure and lacked consideration of population, economic development, and environmental variables. The present study considers these factors and will help to reveal the influence mechanism of carbon emissions. Li combined all influencing factors and put all factors into the model for analysis, which can make the accuracy and stability of the results suffer [27]. We use the GRA algorithm to eliminate minor variables and optimize the model structure.

## 6. Conclusions

This paper takes the four provinces in the Yangtze River Delta region as the research object and uses the combination of GRA and the improved STIRPAT model to mine and analyze the influencing factors of energy carbon emissions in the four provinces, drawing the following conclusions:

1. Although they are all located in the Yangtze River Delta region, the energy consumption structure of the four provinces has shown diversified characteristics in the past 15 years and the total energy carbon emissions have varied significantly. At the same time, due to the differences in population size, economic development level, and industrial structure among provinces and regions, the differences in carbon dioxide emissions between different provinces and regions will be more significant in the future.
2. Based on the existing research results and the actual situation of this paper, 13 influencing factors are determined as candidate sets and the critical factors are screened through the GRA algorithm. The results show that all factors have a certain influence on the energy carbon emissions of each province: the lowest coefficient is 0.5833, the highest is 0.9630, and there are 18 high correlation coefficients (carbon emission factors) higher than 0.9. According to the screening criteria, there are five critical actors in Anhui Province: urbanization rate (P_2_), per capita GDP (A_1_), proportion of secondary industry (A_2_), carbon emission intensity (T_2_) and per capita disposable income (E_3_); Jiangsu: total population (P_1_), urbanization rate (P_2_), proportion of tertiary industry (A_3_), foreign direct investment (A_4_), energy intensity (T_1_) and number of industrial enterprises above scale (E_1_) six critical factors. The five in Shanghai are: total population (P_1_), urbanization rate (P_2_), proportion of tertiary industry (A_3_), energy intensity (T_1_) and SO_2_ emissions (E_2_); Zhejiang also has six critical factors: total population (P_1_), urbanization rate (P_2_), proportion of tertiary industry (A_3_), foreign direct investment (A_4_), carbon emission intensity (T_2_) and per capita consumption expenditure (A_4_). The above factors are most closely related to regional carbon emissions in their respective modules and relevant departments need to focus on achieving peak carbon emission and carbon neutrality goals.
3. Using the factors screened based on GRA, an improved STIRPAT model of each province was constructed and the critical factors were further studied and analyzed. The ranking of the influence of all critical factors in each province is: Anhui: A_2_ > P_2_ > T_2_ > E_3_ > A_1_; Jiangsu: P_1_ > P_2_ > A_3_ > A_4_ > E_1_ > T_1_; Shanghai: P_2_ > P_1_ > A_3_ > T_1_ > E_2_; Zhejiang: P_2_ > E_4_ > A_3_ > A_4_ > P_1_ > T_2_. The most influential factors in Anhui, Jiangsu, Shanghai and Zhejiang are: A_2_, P_1_, P_2_, P_2_; for every 1% positive change in these factors, the carbon emissions of the province will increase by 0.5748%, 1.1761%, 1.1831% and 1.2537%, respectively. The critical influencing factors and corresponding influences of energy carbon emissions in different provinces and regions are different, showing diversity, and the population as a factor has become the module with the highest influence weight and requires further attention.

## 7. Policy Suggestions

Under the background of China’s peak carbon emission and carbon neutrality goals and the development of the Yangtze River Delta regional integration as a national strategy, it is necessary to accelerate the transformation of the economic development mode to low-carbon and intensive, accelerate the green transformation of new energy, promote ecological compensation and industrial chain upgrading and establish a new mechanism for integrated development in terms of ecological compensation, industrial chain upgrading, and public services to promote sustainable and high-quality development in the Yangtze River Delta region. According to our empirical study, the factors influencing carbon emissions in different regions within the region are different and regions should not blindly learn from each other in the process of formulating carbon emission reduction policies. Therefore, by combining the four modules and conclusions of this study, optimization the strategy of energy consumption and carbon emission in the four provinces of the Yangtze River Delta can be put forward:

1. Reasonable control of population size and optimization of population distribution. From the previous experimental results, it is clear that the population factors are the most significant module affecting regional carbon emissions. Shanghai, Zhejiang, and Jiangsu, in particular, should give priority to population growth and the level of urbanization, make use of modern information technology such as big data to predict talent demand, and optimize the allocation of human resources space through talent and industrial policies. Meanwhile, it is necessary to strengthen the guidance and arrangement of the flow of rural population and migrants to cities and promote the two-way flow of urban and rural population, reconstruct urban space by optimizing urban population distribution and industrial layout, actively promote “green buildings”, build “ecological houses”, and advocate green and energy-saving urbanization.
2. Alleviate the contradiction between energy structure transformation and economic development and improve the structure of FDI inflows. The empirical results show that, except for Zhejiang, the economic restructuring of the other three provinces still exacerbates the increase in carbon emissions, indicating that the economic structure has not undergone a fundamental transformation. Therefore, all localities need to strengthen the coordination of economic development and energy structure, achieve a balance between energy supply and demand, improve related supporting construction, and strengthen the coordination and coupling of energy and infrastructure construction. It is worth noting that Anhui and Jiangsu need to pay attention to the effect of industrial restructuring, to encourage the participation of subjects at all levels in the formulation of low-carbon related laws and regulations, and to strengthen supervision. The impact of FDI on carbon emissions in Jiangsu and Zhejiang is still significant. Possible reasons are that local governments are concerned that implementing these policies will hurt the local economy and are not enforcing FDI inflow policies well. Therefore, it is recommended that as many high-carbon industries as possible be included in the FDI ban on inflows, and that FDI-related policies be used in conjunction with other industrial policies.
3. Strengthen scientific and technological research and improve energy efficiency. Technological progress is the key to achieving the peak carbon emission and carbon neutrality goals; various regions can exchange and cooperate at the technical level and rely on the advantages of the high level of scientific research in the Yangtze River Delta to focus on the development of the digital economy and platform economy [61]. To improve the efficiency of energy development and utilization, the actual needs of low-carbon development need to be taken as the guide to strengthen independent innovation of low-carbon technologies. Shanghai has achieved the goal of carbon emission reduction by moving out some high energy consumption enterprises in the past few years, but it could also lead to a decline in economic growth and even economic recession. It is recommended that the innovative development of high energy consumption industries be an important breakthrough for carbon emission reduction in the future. The FDI policies in Jiangsu and Zhejiang are also inseparable from the technical level, and it is recommended that those high-carbon industries be reformed technologically as soon as possible to improve energy efficiency. At the same time, guide FDI into high-tech industries, the introduction of foreign advanced technology is also a major direction.
4. Promote green development and green and low-carbon lifestyles. SO_2_ emission has become one of the critical factors in Shanghai. Therefore, Shanghai must strengthen environmental monitoring, motivate enterprises to actively fulfill their environmental responsibility for carbon reduction, and increase the frequency of environmental information disclosure. Furthermore, the empirical results show that people’s consumption and expenditure are highly correlated with carbon emissions, and per capita consumption expenditure has become a critical factor affecting carbon emissions in Zhejiang Province. Therefore, it is necessary to guide citizens to establish the concept of a green and low-carbon life and to promote it from the government level through major Internet platforms by encouraging participation in green volunteer services and making green consumption, green travel, and green life part of people’s conscious actions.

## Figures and Tables

**Table 1:** Energy Conversion Standard Coal Reference Coefficient and Carbon Emission Coefficient.

Energy Type	Standard Coal Conversion Coefficient	Carbon Emission Coefficient
Coal	0.7143 kgcc/kg	0.7559 kgCO_2_/kg
Coke	0.9714 kgcc/kg	0.8550 kgCO_2_/kg
Crude Oil	1.4286 kgcc/kg	0.5857 kgCO_2_/kg
Gasoline	1.4714 kgcc/kg	0.5538 kgCO_2_/kg
Kerosene	1.4571 kgcc/kg	0.5714 kgCO_2_/kg
Diesel Oil	1.4714 kgcc/kg	0.5921 kgCO_2_/kg
Fuel Oil	1.4286 kgcc/kg	0.6185 kgCO_2_/kg
Gas	1.3300 kgcc/kg	0.4483 kgCO_2_/kg

**Table 2:** Indicator Description of Carbon Emission Influencing Factors.

Target Layer	Indicator Layer	Variable	Description of Independent Variables
Population factors	Total population	P_1_	10,000 people
	Urbanization rate	P_2_	Proportion of urban population in total population (%)
Economic development	Per capita GDP	A_1_	GDP to total population ratio (¥/person)
	Proportion of secondary industry	A_2_	Proportion of added value of secondary industry in GDP (%)
	Proportion of tertiary industry	A_3_	Proportion of added value of tertiary industry in GDP (%)
	Foreign direct investment	A_4_	Total foreign direct investment (10,000$)
Technological factors	Energy intensity	T_1_	Standard energy consumption per unit GDP (%)
	Carbon emission intensity	T_2_	Carbon dioxide emissions per unit of GDP (%)
Other factors	Number of industrial enterprises above scale	E_1_	Industrial enterprises with annual revenue of more than 20 million ¥ (individual)
	SO_2_ emissions	E_2_	10,000 t
	Per capita disposable income	E_3_	¥
	Per capita consumption expenditure	E_4_	¥
	Civil vehicle ownership	E_5_	Vehicle

**Table 3:** Improved STIRPAT Model of four provinces.

Province	Improved STIRPAT Model
Anhui	${\mathrm{lnI}}_{1}{=\mathrm{lna}}_{1}{+b}_{1}{\mathrm{lnP}}_{2}{+c}_{1}{\mathrm{lnA}}_{1}{+d}_{1}{\mathrm{lnA}}_{2}{+e}_{1}{\mathrm{lnT}}_{2}{+f}_{1}{\mathrm{lnE}}_{3}$
Jiangsu	${\mathrm{lnI}}_{2}{=\mathrm{lna}}_{2}{+b}_{2}{\mathrm{lnP}}_{1}{+c}_{2}{\mathrm{lnP}}_{2}{+d}_{2}{\mathrm{lnA}}_{3}{+e}_{2}{\mathrm{lnA}}_{4}{+f}_{2}{\mathrm{lnT}}_{1}{+g}_{2}{\mathrm{lnE}}_{1}$
Shanghai	${\mathrm{lnI}}_{3}{=\mathrm{lna}}_{3}{+b}_{3}{\mathrm{lnP}}_{1}{+c}_{3}{\mathrm{lnP}}_{2}{+d}_{3}{\mathrm{lnA}}_{3}{+e}_{3}{\mathrm{lnT}}_{1}{+f}_{3}{\mathrm{lnE}}_{2}$
Zhejiang	${\mathrm{lnI}}_{4}{=\mathrm{lna}}_{4}{+b}_{4}{\mathrm{lnP}}_{1}{+c}_{4}{\mathrm{lnP}}_{2}{+d}_{4}{\mathrm{lnA}}_{3}{+e}_{4}{\mathrm{lnA}}_{4}{+f}_{4}{\mathrm{lnT}}_{2}{+g}_{4}{\mathrm{lnE}}_{4}$

**Table 4:** Table Models Summary.

Model	R	R-Square	Adjusted R-Square	Standard Error in Estimation
Anhui	0.9936	0.9872	0.9801	0.3611
Jiangsu	0.9859	0.9721	0.9513	0.4343
Shanghai	0.8371	0.7007	0.5344	0.3866
Zhejiang	0.8696	0.7562	0.5734	0.0743

**Table 5:** Anhui Model Test Results.

Variable	Unstandardized Coefficient	Standard Error	Standard Coefficient	t-Statistic
Constant coefficient	9.0179	0.0970	0.0000	92.9510
InP_2_	0.3804	0.0208	0.2356	18.3165
InA_1_	0.1025	0.0040	0.2392	25.3263
InA_2_	0.5748	0.0936	0.1925	6.1437
InT_2_	−0.1341	0.0107	−0.1860	−12.5380
InE_3_	0.1187	0.0051	0.2334	23.4321

**Table 6:** Jiangsu Model Test Results.

Variable	Unstandardized Coefficient	Standard Error	Standard Coefficient	t-Statistic
Constant coefficient	0.6335	1.3133	0.0000	0.4823
InP_1_	1.1761	0.0736	0.2465	8.1667
InP_2_	0.5128	0.0437	0.2646	6.9673
InA_3_	0.3225	0.0337	0.2025	9.5825
InA_4_	0.2094	0.0437	0.2646	4.7893
InT_1_	−0.0571	0.0184	−0.1040	−3.1009
InE_1_	−0.0593	0.0534	−0.0574	−1.1111

**Table 7:** Shanghai Model Test Results.

Variable	Unstandardized Coefficient	Standard Error	Standard Coefficient	t-Statistic
Constant coefficient	6.8413	0.6913	0.0000	9.8962
InP_1_	0.4481	0.9175	0.7273	4.8837
InP_2_	1.1831	0.6837	0.2984	1.7304
InA_3_	0.0445	0.0512	0.8939	0.8696
InT_1_	−0.0111	0.0127	−0.0767	−0.8751
InE_2_	0.0034	0.0068	0.0868	0.4960

**Table 8:** Zhejiang Model Test Results.

Variable	Unstandardized Coefficient	Standard Error	Standard Coefficient	t-Statistic
Constant coefficient	9.6033	0.8957	0.0000	10.7216
InP_1_	0.0640	0.0766	0.0457	0.8358
InP_2_	1.2537	0.4640	0.4610	2.7021
InA_3_	−0.1277	0.1250	−0.1184	−1.0216
InA_4_	0.0980	0.0714	0.2388	1.3735
InT_2_	−0.0018	0.0195	−0.0063	−0.0930
InE_4_	0.5603	0.0186	0.1881	3.0095

## Data Availability

The data used to support the findings of this study are available from the corresponding author upon request.
